# Author Correction: Role of the particle size polydispersity in the electrical conductivity of carbon nanotube-epoxy composites

**DOI:** 10.1038/s41598-018-25538-x

**Published:** 2018-05-09

**Authors:** Maryam Majidian, Claudio Grimaldi, László Forró, Arnaud Magrez

**Affiliations:** 1 Laboratory of Physics of Complex Matter, Ecole Polytechnique Fédérale de Lausanne, Station 3, CH-1015 Lausanne, Switzerland; 2 Crystal Growth Facility, Ecole Polytechnique Fédérale de Lausanne, Station 3, CH-1015 Lausanne, Switzerland

Correction to: *Scientific Reports* 10.1038/s41598-017-12857-8, published online 02 October 2017

The Supplementary Information file originally published with this Article was incorrect. The Supplementary Information file that now accompanies the Article is correct.

