# Supplementary material for: Role of the particle size polydispersity in the electrical conductivity of carbon nanotube-epoxy composites
Source: Sci Rep. 2017 Oct 2;7:12553. doi: 10.1038/s41598-017-12857-8 (PMC5624922; doi:10.1038/s41598-017-12857-8)
Supplement: Supplementary file 1 — Supplementary info [file 41598_2017_12857_MOESM1_ESM.pdf]

Supplementary Information

Role of the particle size polydispersity in the electrical conductivity of carbon nanotube-epoxy composites

Maryam Majidian[a], Claudio Grimaldi[a] László Forró[a], Arnaud Magrez[a,b]

[a] Laboratory of Physics of Complex Matter, Ecole Polytechnique Fédérale de Lausanne, Station 3, CH-1015 Lausanne, Switzerland

[b] Crystal Growth Facility, Ecole Polytechnique Fédérale de Lausanne, Station 3, CH-1015 Lausanne, Switzerland

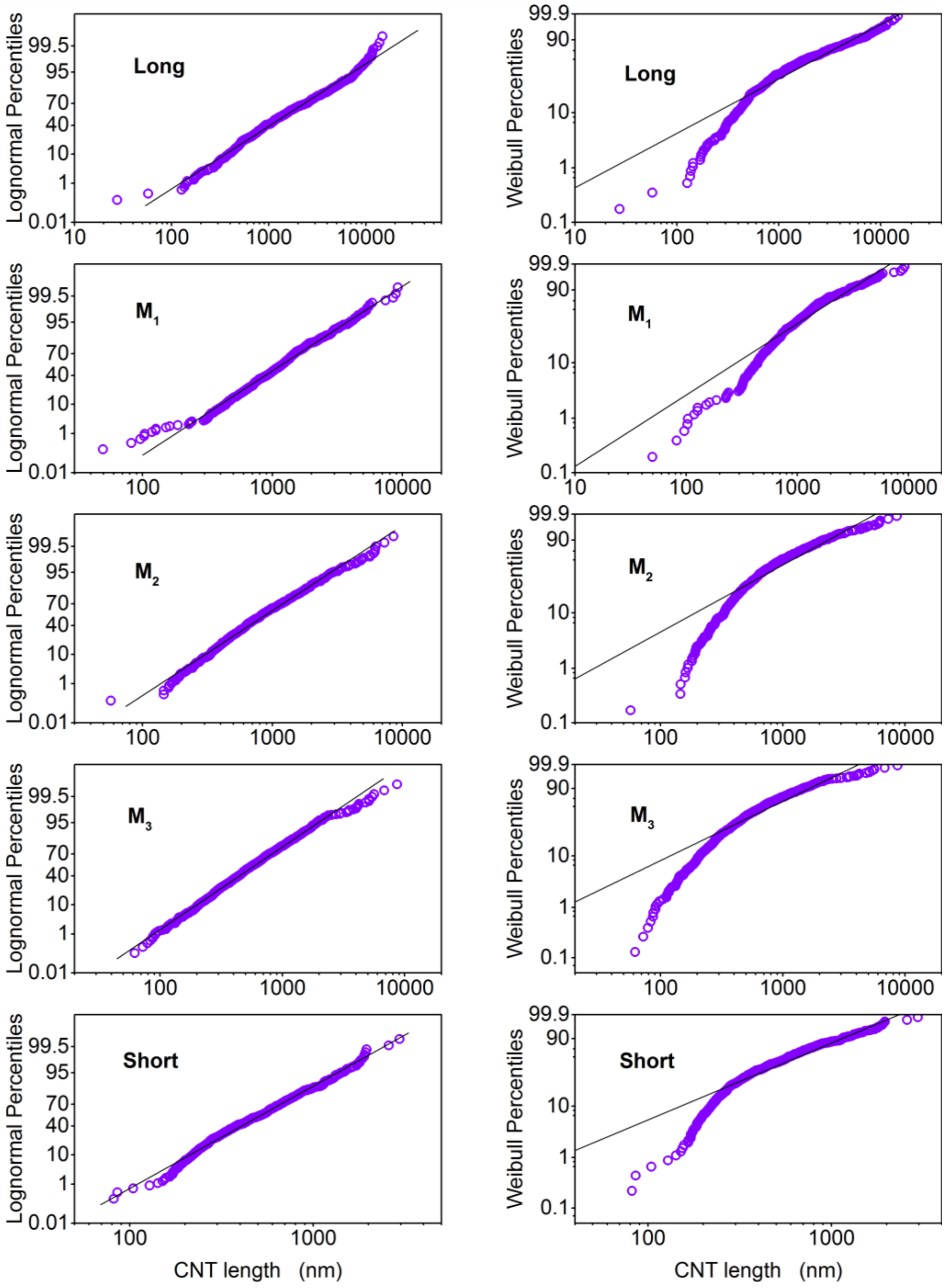

Supplementary Figure 1. Probability plots of the measured CNT lengths for five different batches obtained from chosen combinations of the milling time and the rotational speed. The probabilities are plotted assuming a lognormal distribution (left column) and a Weibull distribution (right column). The solid lines indicate the corresponding theoretical probabilities.

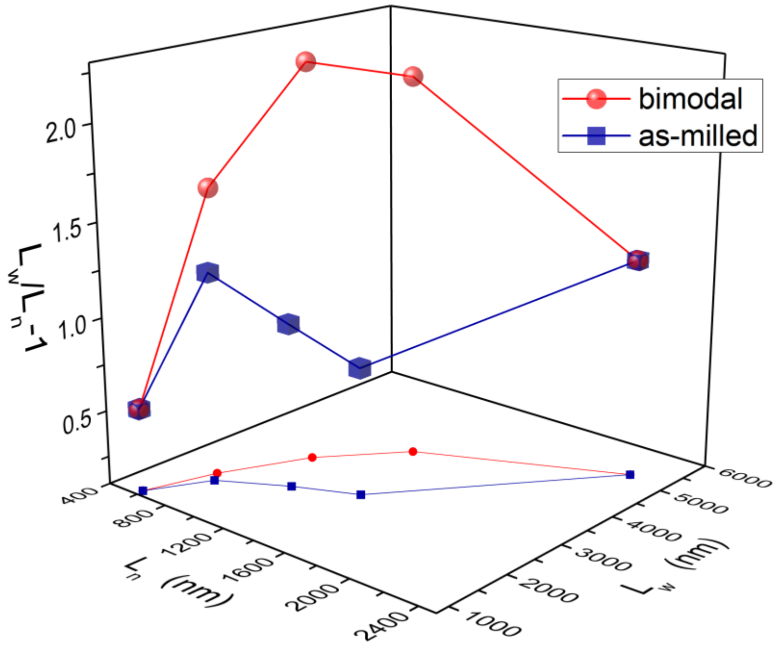

Supplementary Figure 2. Scaled variance $\langle L^2 \rangle / \langle L \rangle^2 - 1 = L_w / L_n - 1$ for the unimodal (as-milled) and bimodal CNTs.

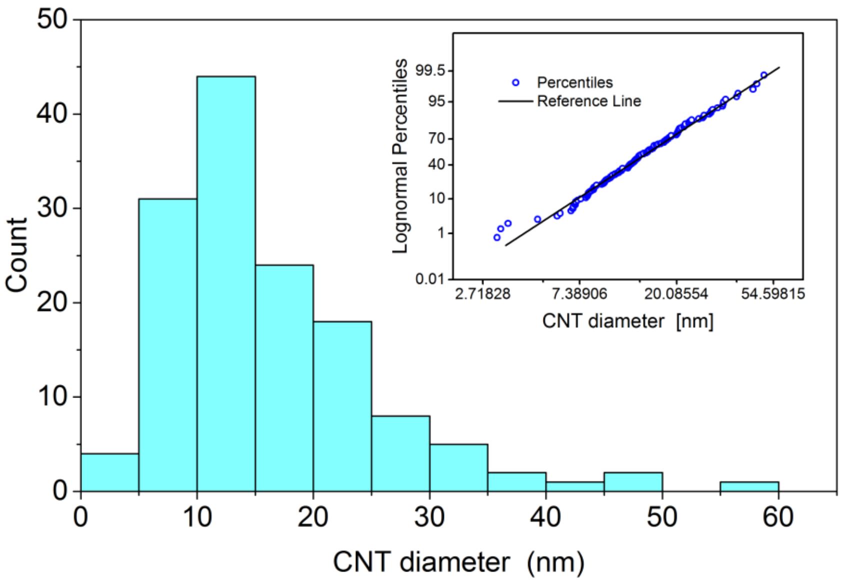

Supplementary Figure 3. Histogram of the CNT diameters D obtained from 140 values measured from TEM images of microtome slices of CNT-SU8 composites. The first and second moment of D extracted from the measured diameters are, respectively, $\langle D \rangle \approx 16$ nm and $\langle D^2 \rangle \approx 346$ nm^2. Inset: lognormal probability plot.

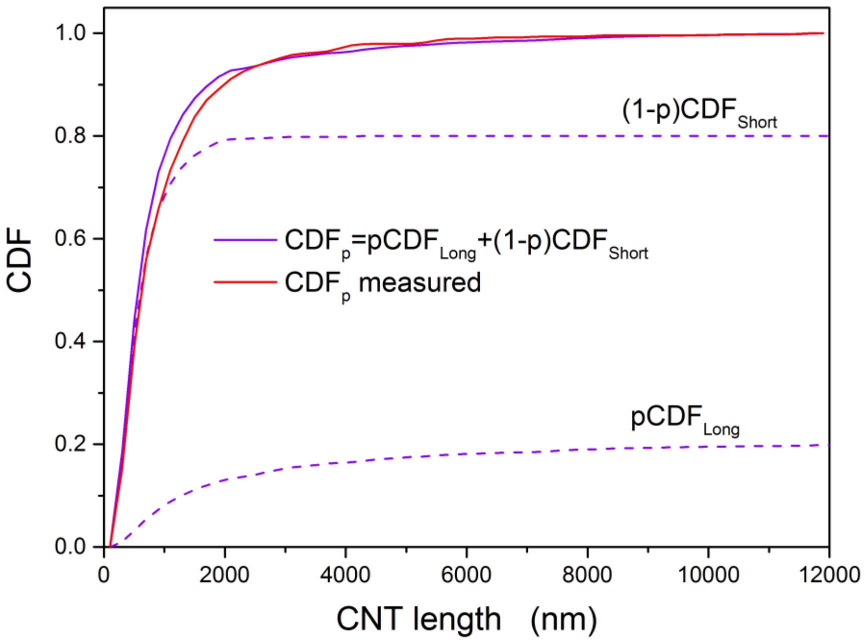

Supplementary Figure 4. Comparison between the calculated and the measured cumulative distribution functions (CDFs) of the bimodal distribution of CNT lengths for p=0.2. The dashed curves are the partial contributions of the measured CDFs of the Long and Short batches.

Supplementary notes 1

We define nanotubes of type i those wormlike cylinders that have length L_i and diameter D_i. A pair of nanotubes is considered as connected if the distance between their closest surfaces is smaller than a given distance δ. The probability p_{ij} that a nanotube of type j is connected to a given nanotube of type i is given by equation (7) of the main text. Next, we define the multicomponent degree distribution of a nanotube of type i as the probability $P_i(1,k_1;2,k_2;\cdots)$ that the nanotube is connected to k_1 nanotubes of type 1, k_2 nanotubes of type 2, and so on. Assuming that spatial correlation is weak, the multicomponent degree distribution can be written as a product of binomial distributions, $P_i(1,k_1;2,k_2;\cdots)=\prod_j P_{ij}(k_j)$, each of which gives the probability that a nanotube of type i is connected precisely with k_j nanotubes of type j:

$$P_{ij}(k_j)=\binom{N_j-\delta_{ij}}{k_j}p_{ij}^{\,k_j}(1-p_{ij})^{N_j-\delta_{ij}-k_j}, \qquad (1)$$

where N_j is the number of nanotubes of type j and $\delta_{ij}=1$ if $i=j$ and $\delta_{ij}=0$ otherwise. We take the limit of large volume such that N_i/V remains finite for all i. Equation (1) reduces in this way to a Poisson distribution:

$$P_{ij}(k_j)=\frac{Z_{ij}^{\,k_j}}{k_j!}e^{-Z_{ij}}, \qquad (2)$$

where $Z_{ij}=Nx_jp_{ij}$ is the average number of nanotubes of type j that are connected to a given nanotube of type i, N is the total number of nanotubes, and $x_j=N_j/N$.

For nanotubes of large aspect ratio, closed loops of connected nanotubes are statistically irrelevant. The topology of the network formed by connected nanotubes is therefore tree-like. In this case, the mean size S of finite components of connected particles can be calculated easily. We start by considering that a randomly selected node of the network has probability x_i of being occupied by a nanotube of type i, and that it is connected to k_j other nodes occupied by nanotubes of type j. The mean size S_i of the cluster to which the selected node belongs is therefore

$$S_i=x_i+x_i\sum_j\sum_{k_j}k_jP_{ij}(k_j)T_j=x_i+x_i\sum_jZ_{ij}T_j, \qquad (3)$$

where we have made use of equation (2) and T_j is the mean cluster size of one of the branches attached to the selected node. T_j is given by the sum of the mass (unity) of one neighbor of the selected node and the mean size of each of the remaining subbranches connected to the neighbor:

$$T_j=1+\sum_l\sum_{k_l}k_lP_{jl}(k_l)T_l=1+\sum_lZ_{jl}T_l\,. \qquad (4)$$

Finally, the mean cluster size is given by $S=\sum_i x_iS_i$. The percolation threshold is given by value of δ (encoded in the definition of Z_{ij}) such that S diverges.
